# The development of two novel species-specific primers for identifying genus *Crassostrea*, with focus on *C. sikamea* and *C. ariakensis*

**DOI:** 10.1080/23802359.2018.1501299

**Published:** 2018-08-27

**Authors:** Taoni Wang, Weiming Quan, Qiqun Cheng, RuiLang Fan

**Affiliations:** a Key Laboratory of Oceanic and Polar Fisheries, Ministry of Agriculture, East China Sea Fisheries Research Institute, Chinese Academy of Fishery Sciences, Shanghai, China;; b College of Fisheries and Life Science, Shanghai Ocean University, Shanghai, China

**Keywords:** Reef-forming oyster, *Crassostrea sikamea*, *C. ariakensis*, larvae identification, ecological restoration

## Abstract

Oyster reefs have important ecological functions and environmental service values, such as filtering water, providing fish habitats, maintaining biodiversity, and carbon sequestration. Oyster reef recovery has become one of the most important means for fishery resource conservation and ecosystems restoration of coastal regions*. C. sikamea* and *C. ariakensis* are two important reef oysters along the coast of China. However, it is very difficult to identify the larvae of these two oysters. To solve this problem, we developed two pairs of novel species-specific PCR primers for identifying these two oysters. The forward *C. sikamea*-specific primer is Cs-F: 5′-CGAAGAGGGGCATGATAAATGAGG-3′, and the reverse primer is Cs-R: 5′-ATATGAACTTCTCCAACCTCCCC-3′, both of them within the scope of mitochondrial ND5 gene. The forward *C. ariakensis*-specific primer is Ca-F: 5′-GGGCAAATAAAAGGCAAAACCC-3′, located between tRNA^ser^ and tRNA^pro^, and the reverse primer is Ca-R: 5′-CATAAACTTCTGCAAGACTCCC-3′, lies between tRNA^pro^ and small subunit ribosomal RNA (ssrRNA). These two primers can identify the larvae of *C. ariakensis* and *C. sikamea* from other oysters rapidly and effectively, with extremely high accurate rate (=100%). This molecular identification method does not require prior sorting of larvae. These two specific primers are good contribution to molecular identification of *C. sikamea*, *C. ariakensis* and other oysters, and could be a useful tool for oyster larvae identification and ecological restoration.

Oysters (Ostreidae, Pterioida) are widely distributed in temperate estuaries and coastal areas (Beck et al. 2011). Oysters are economically important species and aquaculture shell-fishes with the largest products in the world. More than 100 species are found in the world and about 20 species in China (Wang and Wang 2008). Oyster reefs also have important ecological functions and environmental service values, such as filtering water (Nelson et al. 2004; Quan et al. 2007), providing fish habitats (Coen et al. 1999; Quan et al. 2009, 2012, 2013), maintaining biodiversity (Quan et al. 2006, 2011, 2013; Rodney and Paynter, 2006), and carbon sequestration (Shen et al. 2011). Oysters are ‘keystone species’ of the oyster reef ecosystem. Among all the oysters distributed globally, six species, i.e. *C. sikamea* (Quan et al. 2012) , *C. ariakensis* (Quan et al. 2009, 2011), *C. gigas* (Lejart and Hily. 2011), *C. virginica* (Boudreaux et al. 2006), *Ostrea lurida* (Brumbaugh and Coen. 2009), *O. edulis* (Smyth and Roberts. 2010), are reported as reef-building species. In China, five oysters, i.e. *C. sikamea, C. ariakensis*, *C. gigas*, *C. hongkongensis*, and *C. angulata* are common species and two of them, i.e. *C.sikamea* and *C.ariakensis*, are reef-forming species.

Identifying the reef-forming species accurately is an important pre-requisite for analyzing the spatial niche and reef-building ability of oysters, inspecting oyster competition for adhesion substrates space use, and colonizing ability. Due to the diverse habitat and shell plasticity, external morphological characteristics of oysters often changes greatly with the different living environment. Distinguishing them relying only on shell exterior appearance is difficult.

The life cycle of oysters consist of a larval phase (prior to settlement) and adult phase (after settlement). Once settled on the required hard substrate, adults cannot relocate (Coen and Humphries 2017). Based on successful recruitment of oyster larvae and later larvae in successive years, oyster reefs can continue to expand (Coen and Luckenbach 2000; Luckenbach et al. 2005). Reliably detecting larvae during critical life stages is urgent to forecast population recruitment.

Larvae cannot be reliably identified using conventional morphologic observations similar to the larvae of most marine organisms (Christo et al. 2010). This method is problematic during earlier stages of larval development and relies on proficient expertise on bivalve larvae identification. Although traditional microscopic screening based on morphology is possible, it is time-consuming, laborious, and need skilled expertise (Boudry et al. 2003).

Molecular probes and markers are widely used for the identification of oyster species in the last decades (Morgan and Rogers 2001; Anil et al. 2002). For example, using molecular markers, Anderson and Adlard (1994) and Kenchington et al. (2002) classified the oyster populations described in different geographical regions into one species, Hedgecock et al. (1999) verified a species’ presence in a geographic area, and various *Crassostrea* oysters species were distinguished (Banks et al. 1993, O’Foighil et al. 1995, Klinbunga et al. 2000, 2001, 2003, Boudry et al. 2003). In order to solve difficulty in the identification of larvae oysters, especially the larvae of two main reef-forming oysters, i.e. *C. sikamea* and *C. ariakensis*, in China, we developed two pairs of species-specific primers. These two pairs of primers will be widely used for the identification of the larvae of *C. sikamea* and *C. ariakensis*, and can also be helpful to improve the detection level of period of larval release and settlement in complex environmental and water samples.

Adult individuals of five oysters species including *C. sikamea*, *C. ariakensis*, *C. gigas*, *C. angulata*, and *C. hongkongensis*, were collected from the intertidal and coastal zones of China, i.e Dalian(DL), Weihai(WH), Nantong(NT), Shanghai(SH), Ningbo(NB), Ningde(ND), and Yangjiang(YJ). The adductor tissues of these individuals were stored at −20 °C until used. *C. sikamea* and *C. ariakensis* larvae were collected from Liyashan, Luchao, and Xiangshan, using a 200-mesh sieve. They were preserved in 95% ethanol fixative and stored at 4 °C until extracted. All specimens were deposited in East China Sea Fisheries Research Institute.

Genomic DNA of adult adductor muscles (about 20mg) were extracted using the TIAN & Marine Animal DNA kit (Tiangen). The product contents of this DNA extraction kitinclude Buffer GA, Buffer GB, Buffer GD, Buffer PW, Buffer PW, Buffer PW, Buffer PW, ProteinaseK, and adsorption Column CB3 (Spin Column CB3), CollectionTube 2ml. Larvae samples were concentrated by vacuum filtration through a 0.45 micron nitrocellulose filter or isolated under an Optical microscope were transferred directly to 1.5 ml PCR tubes immersed in ethanol, and DNA-extracted using the Marine animal tissue genomic DNA extraction kit (Qiagen) following the suppliers instructions. DNA was retrieved in 100 μl of elution buffer and stored at 4 °C.

Amplification and sequencing of the 16SrDNA markers was carried out using the universal primers 16SAR/BR. Approximately 400–600bp amplicons correspond to mitochondrial genome base sites between 1865 and 2365 of *C. sikamea* (accession number FJ841966.1) and from 1870 to 2359 of *C. ariakensis* (accession number FJ841964.1). Standard PCR reactions were performed using a 25 μL volume of PCR mixture, containing DNA Template 1 μL, 1 μL of each primer, 2 × Master Mix 12.5μL (0.1U/μL Taq DNA polymerase; 4mm MgCl_2_; 0.4 mM dNTPs), and ddH_2_O 9.5 μL. PCR amplifications were performed under the following conditions: initial pre-denaturation at 94 °C for 5 min followed by 35 thermal cycles of denaturation at 94 °C for 30 s, annealing at 52 °C for 45 s, and extension at 72 °C for 1 min. A final extension was performed at 72 °C for 7 min.

Two pairs of primers were designed by comparing complete mitochondrial sequences of *C. sikamea* and *C. ariakensis* with other members of the family *Crassostrea* including those of *C. gigas*, *C. hongkongensis*, and *C. angulata*. The forward *C. sikamea*-specific primer is Cs-F: 5′-CGAAGAGGGGCATGATAAATGAGG-3′, and the reverse primer is Cs-R: 5′-ATATGAACTTCTCCAACCTCCCC-3′, both of them within the scope of mitochondrial ND5 gene. The forward *C. ariakensis*-specific primer is Ca-F: 5′-GGGCAAATAAAAGGCAAAACCC-3′, located between tRNA^ser^ and tRNA^pro^, and the reverse primer is Ca-R: 5′-CATAAACTTCTGCAAGACTCCC-3′, lies between tRNA^pro^ and small subunit ribosomal RNA (ssrRNA).

The PCR reaction components and amplification conditions of these two specific primers are the same. PCR reactions were performed using a 20 μl volume of PCR mixture containing 1 μl of each primer, 2 × Master Mix 10 μl (0.1 units/μl Taq DAN polymerase; 4m MMgCl2; 0.4 mM dNTPs), ddH20 7.5 μl, and Template 0.5 μl. PCR amplifications were performed under the following conditions: initial pre-denaturation at 95 °C for 3 min followed by 35 thermal cycles of denaturation at 95 °C for 15 s, annealing at 55 °C for 15 s, and extension at 72 °C for 15 s. A final extension was performed at 72 °C for 3 min. In order to control the contamination, high-temperature sterilization is required for each pipette tips with all PCR solutions, and negative control reactions were performed with sterilized water and other oyster species. Verification of the species-specific PCR results of *C. sikamea* and *C.ariakensis* were undertaken with a DNA marker DL500 using 2μL of each PCR product electrophoresed separately in 3% agarose gel containing ethidium bromide.

The specificity of the predicted *C. sikamea*-specific primers and *C. ariakensis*-specific primers was empirically tested by PCR amplification of genomic DNA of the five *Crassostrea* species using sterile water as negative control. For *C. sikamea* specific primers, the expected 114 bp amplicon can only be amplified from *C. sikamea* individuals, while the other four oysters and sterile water had no amplicons and only *C. ariakensis* can obtain the expected 126 bp target fragment (Figure 1(a,b)).

**Figure 1:**
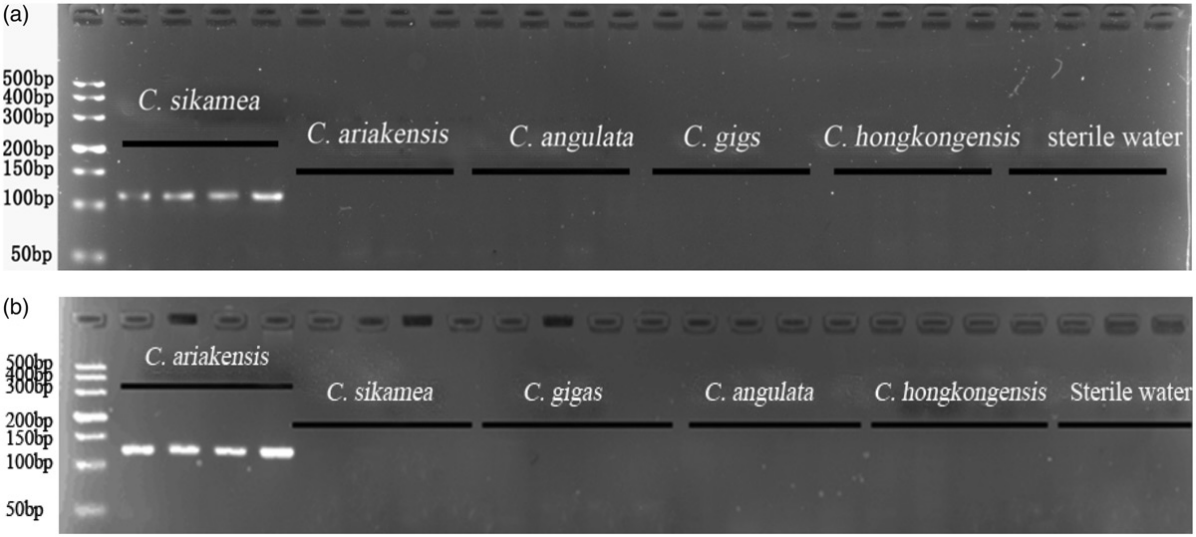
The agarose gel electrophoresis image of five oysters PCR products. Notes: (a) from left to right: lane 1, DL500 marker; lanes 2–5, *C. sikamea*; lanes 6–9, *C.ariakensis*; lanes 10–13, *C. angulata*; lanes 14–17, *C. gigas*; lanes 18–21, *C. hongkongensis*; lanes 22–24, sterile water (negative control); (b) from left to right: lane 1, DL500 marker; lanes 2–5, *C.ariakensis*; lanes 6–9, *C. sikamea*; lanes 10–13, *C. gigas*; lanes 14–17, *C. angulata*; lanes 18–21, *C. hongkongensis*; lanes 22–24, sterile water (negative control).

The PCR product was sequenced by Sanger’s dideoxy chain termination method and then compared with Vector NTI software. The results indicate that the forward *C. sikamea*-specific primer Cs-F and the reverse primer Cs-R are both within the scope of mitochondrial ND5 gene, but the forward *C. ariakensis*-specific primer Ca-F is located between tRNA^ser^ and tRNA^pro^ and the reverse primer Ca-R lies between tRNA^pro^ and small subunit ribosomal RNA(ssrRNA).

**Larvae sample testing:** It was possible to amplify the isolated larvae as templates by using *C. sikame*a-specific primers and *C. ariakensis*-specific primers. Detection accuracy was 100% when larvae or spat were used as templates (Figure 2(a,b)). Identifying oyster larvae rapidly and accurately is of great importance for the seashore ecological management because it is not only useful to judge natural distribution of the larval across the areas of the sea, settlement, and recruiting commercial fisheries, but also to monitor the presence of other new oysters in the native ecosystem (Bax et al. 2005). Above all, identifying *C. sikamea* and *C. ariakensis* larvae in China is particularly important because it can provide scientific guidance for further analysis about the presence/absence and spatial niche of these two oysters distributed in water body, evaluating reef-forming ability of oysters, and monitoring the ecological status of seashore.

**Figure 2.**
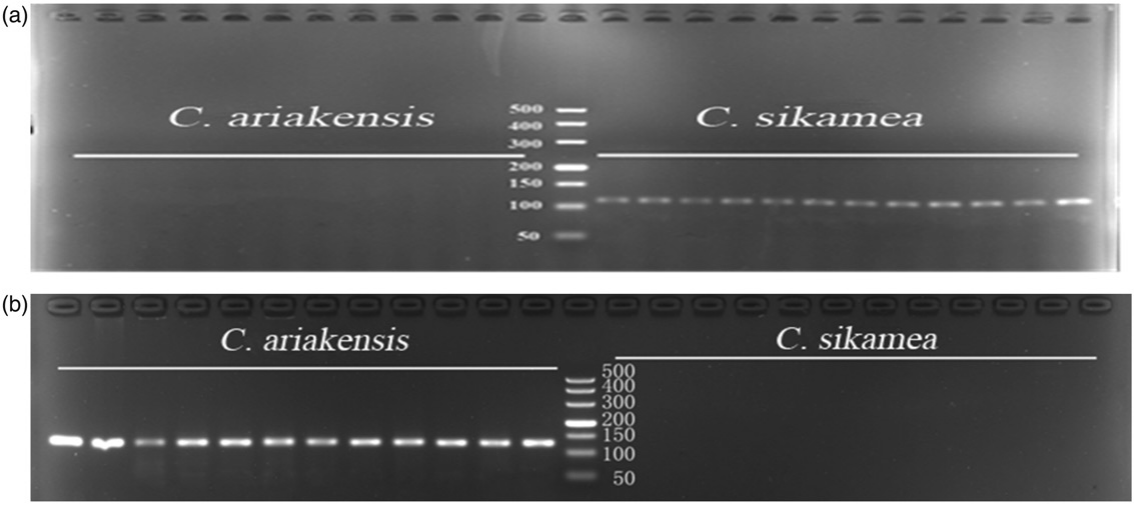
The PCR products agarose gel electrophoresis image of *C. ariakensis* and *C. sikamea*. (a) and (b) from left to right: lanes1-12, *C.ariakensis*; lane 13, DL500 marker; lanes 14–25, *C. sikamea*
